# Attenuated impression of irony created by the mismatch of verbal and nonverbal cues in patients with autism spectrum disorder

**DOI:** 10.1371/journal.pone.0205750

**Published:** 2018-10-15

**Authors:** Simon Nuber, Heike Jacob, Benjamin Kreifelts, Anne Martinelli, Dirk Wildgruber

**Affiliations:** 1 Department of Psychiatry and Psychotherapy, University Hospital Tübingen, Tübingen, Germany; 2 Department of Child and Adolescent Psychiatry, University Hospital Frankfurt, Frankfurt am Main, Germany; Department of Psychiatry and Neuropsychology, Maastricht University Medical Center, NETHERLANDS

## Abstract

Perception of irony has been observed to be impaired in adults with autism spectrum disorder. In typically developed adults, the mismatch of verbal and nonverbal emotional cues can be perceived as an expression of irony even in the absence of any further contextual information. In this study, we evaluate to what extent high functioning autists perceive this incongruence as expressing irony. Our results show that incongruent verbal and nonverbal signals create an impression of irony significantly less often in participants with high-functioning autism than in typically developed control subjects. The extent of overall autistic symptomatology as measured with the autism-spectrum questionnaire (AQ), however, does not correlate with the reduced tendency to attribute incongruent stimuli as expressing irony. Therefore, the attenuation in irony attribution might rather be related to specific subdomains of autistic traits, such as a reduced tendency to interpret communicative signals in terms of complex intentional mental states. The observed differences in irony attribution support the assumption that a less pronounced tendency to engage in higher order mentalization processes might underlie the impairment of pragmatic language understanding in high functioning autism.

## Introduction

Comprehension of figurative language is a momentous aspect of social interaction. Here, figurative language is an umbrella term for all terms, idioms or utterances whose intended meaning differs from their literal meaning [1]. One type of figurative language is irony [2], which is generally defined as “the use of words to express something other than and especially the opposite of the literal meaning” [3]. Irony is often used to fine-tune a message and can influence how positively or negatively an utterance is perceived. For example, it has been shown that criticism expressed ironically is perceived less critically than criticism expressed literally [4]. In addition, the component of humor, which can be created in irony by the mismatch between what is said and what is meant, can lessen the harm to the relationship between speaker and addressee which may be caused by criticism. On the other hand, irony also affects the perception of positive statements, with ironic compliments being perceived less positively than literal ones [4–6].

Several methods have been described to express and recognize irony in an utterance. A common method relies on the contrast between literal meaning and a shared environmental context. Assuming that speaker and addressee are both aware of the context, for example stormy weather, the latter can infer the ironic intention of the utterance “It’s such a nice day!” only from the obvious contradiction between what is said and how the actual environmental conditions are (taking into account common cultural conventions) [7]. An obvious contrast between literal meaning and an environmental context, however, is not always necessary. There is evidence that sarcastic irony, which is used to criticize, offend or mock other people [8], can be conveyed solely by specific prosodic cues such as slower tempo, lower pitch level and greater intensity [9] even in the absence of further contextual information. Moreover, it has been shown that a mismatch between verbal and nonverbal (e.g., facial expression, prosody) emotional cues can be sufficient to create an impression of irony in the listener [10].

There are estimations which suggest that, in contemporary American literature, an ironic expression can be found on every four pages [11], and in American TV-shows, there are eight of these expressions per hour [12]. In conversations among friends, eight percent of utterances are ironic [13]. Due to its very common use, difficulties in perception of irony, as shown in, for example, schizophrenia [14], alcoholism [15] or autism [16–24] have a substantial influence on social interaction competences.

In autism spectrum disorders (ASD) in particular, persistent deficits in social communication and social interaction across multiple contexts are considered to be essential diagnostic criteria [25]. The term spectrum disorder, however, reflects that persons with ASD show a wide interindividual range in severity of the symptomatology, ranging from decreased interest in social communication (despite almost unimpaired communicative abilities), to strong impairments in active vocabulary and in initiation of and response to social contact [25], including impairments of prosody perception [26] and emotional recognition [27]. Furthermore, it should be noted that even typically developed individuals may show mild autistic traits, without reaching the diagnostic criteria for ASD [28–30].

The aim of the present study was to evaluate differences in the impression of irony created by the mismatch of verbal and nonverbal cues in audiovisual stimuli in patients with ASD as compared to typically developed subjects, and to evaluate whether differences in irony perception are associated with severity of autistic symptoms. Previous studies in irony perception in autists showed heterogeneous results. While many of them have reported deficits in irony perception in autists [16–24], some could not show any group differences [31–33]. A possible explanation for the observed variability might lie therein that these studies differed considerably in terms of mentalization demands. While some studies only required first-order mentalization (i.e., assumption about a mental state of another person, such as his current affective state or a specific belief or factual knowledge) [17, 19, 21, 24, 31–33], other tasks required assessments of at least second-order mentalizations (i.e. assumptions about the beliefs another person has about the mental states of others [21]). In this regard it is very interesting to note that significant group differences with impairments in irony perception in ASD subjects have been uniformly observed in all studies that required at least second-order mentalizing processes [16, 18, 22, 23]. Studies requiring only first-order mentalization processes to detect irony (e.g., based on identification of a mismatch between the mentalization of a person’s factual knowledge and their direct statements), however, partly confirmed significant impairments in ASD subjects [17, 19, 21, 24] but partly failed to show clear cut differences between ASD and TD subjects [31–33].

In the current study, dynamic visual and auditory stimuli were combined to evaluate the effects of incongruent verbal and nonverbal emotional cues on the perceived impression of irony using stimuli with a high ecological validity. To this end, we used short videos showing actors who nonverbally convey a positive, negative or neutral emotional state using prosody and facial expression, and at the same time, express their current emotional state verbally. Thereby, nine possible combinations resulted for the final stimulus videos. For the current study, we only considered differences in congruency between verbal and nonverbal signals: congruent stimuli (verbal and nonverbal cues match regarding the expressed emotion, e.g. positive verbal and positive nonverbal expression) and incongruent stimuli (slightly incongruent: a neutral and a positive or negative expression combined, e.g. a neutral verbal and a positive nonverbal expression; strongly incongruent: a positive and a negative expression combined, e.g. a negative verbal with a positive nonverbal expression). These stimulus videos were presented to the participant, who had to categorize their impression of the speaker’s expression (forced choice differentiation between: "angry", "happy", "ironic" or "ambivalent"). In this context, it is important to note that during recording of the stimuli the actors were not instructed to express irony at all. Thus, in the current study we didn’t aim to assess differences in correct identification of certain verbal or nonverbal cues. Instead, we aimed to evaluate the tendency to interpret the mismatch of verbal and nonverbal cues in terms of complex intentional mental states. More specifically, it was assumed that evaluation of the stimuli using predominantly first-order mentalization (e.g. what is the current emotional state of the speaker) would bias responses towards unequivocal emotional categories (“angry”, “happy”) after presentation of congruent stimuli, whereas incongruent stimuli might rather be perceived as expressing mixed feelings (“ambivalent”) under these conditions. The attribution of incongruent verbal and nonverbal emotional cues as expressing irony, however, is expected to require second-order mentalization to some extent (i.e. the belief that the speaker believes that his utterance will be correctly understood as expressing irony by the listener).

Under the assumption that TD subjects show a tendency to interpret communicative signals using first-order and second-order mentalizations, and that the tendency to rely on second-order processes is less pronounced in subjects with ASD, we formulated the following hypotheses:

1. The mismatch between verbal and nonverbal cues was expected to create the impression of irony. More specifically, we hypothesized that incongruent stimuli would be categorized as “ironic” more often than congruent stimuli across both groups.
2. Regarding group differences, we hypothesized that the choice frequency of the “ironic” category would be lower in the ASD group as compared to the typically developed (TD) group. More specifically, we expected the most predominant attenuation of irony attribution in the ASD group for incongruent stimuli.

In a subsequent explorative analysis, we evaluated the impact of the level of incongruence (slightly and strongly incongruent) on the impression of irony and the relationship of irony attribution with the severity of the autistic symptomatology. Corresponding investigations were conducted concerning the reaction times.

## Materials and methods

### Participants

Twenty patients with high functioning ASD (12 men and 8 women; mean age = 33.8 years, *SD* = 8.77 years; age range 20–52 years; 4 with secondary school certificate / apprenticeship, 16 with college/university degree), with a diagnosis of high-functioning early childhood autism (F84.0) or Asperger-Syndrome (F84.5) according to the ICD-10 criteria, were recruited from the special out-patient consultation service for adults with autism-spectrum-disorders of the University Hospital Department of Psychiatry and Psychotherapy Tübingen, where they have been diagnosed on the basis of intense clinical examinations by fully trained psychiatrists. The examinations included a comprehensive anamnesis and evaluation of interactional behavior as well as structured questionnaires completed by participants with ASD (Autism-spectrum Quotient AQ [29], Empathy Quotient EQ [34], Multiple-choice Vocabulary Intelligence Test MWT-B [35], Beck Depression Inventory BDI [36]) and at least one relative (Social Responsiveness Scale SRS [37], Social Communication Questionnaire SCQ/FSK [38], Marburg Rating Scale for Asperger’s Syndrome MBAS [39]) able to report firsthand about the participant’s behavior during the first decade of life. The patients gave their consent to be informed about clinical studies during the diagnostic procedure and were contacted via e-mail. Participants reported having no hearing or vision disorders. The control group comprised twenty TD healthy participants that were individually matched by age, gender and educational background to the participants of the ASD group. None of these twenty persons (mean age = 33.5 years, *SD* = 9.45 years; age range 20–53 years) reported hearing or vision disorders, neurological or psychiatric disorders or medication. There was no significant difference in age between the TD group and the ASD group (*p* = 0.72 in the Mann-Whitney-U-Test). Table 1 shows the participants’ characteristics.

**Table 1:** Participants’ characteristics.

	ASD group	TD group
**Age (range)**	33.8 ± 8.8 (20–52)	33.5 ± 9.5 (20–53)
**Sex distribution**	12 men 8 women	12 men 8 women
**Diagnosis**	High functioning early childhood autism: 5 Asperger syndrome: 15	—
**AQ Total** **Social skills** **Attention switching** **Attention to detail** **Communication** **Imagination**	38.3 ± 7.2 8.0 ± 2.4 8.1 ± 2.4 7.2 ± 1.8 7.6 ± 2.1 7.0 ± 2.3	11.5 ± 4.1 1.2 ± 1.6 4.0 ± 1.1 3.3 ± 1.9 1.2 ± 1.0 2.0 ± 1.1
**EQ**	14.1 ± 8.0	Not tested
**IQ (MWT-B)**	113.7 ± 14.1	Not tested

Numbers indicate means with standard deviations.

### Ethics statement

The study was planned and performed in accordance with the ethical principles of the Declaration of Helsinki (Code of Ethics of the World Medical Association) and was approved by the Ethics Committee of the Faculty of Medicine of the Eberhard Karls University and the University Hospital Tübingen. All participants took part voluntarily and gave their written informed consent prior to inclusion in the study. They received a small financial compensation for their participation.

### Measure of autistic symptomatology

To assess the severity of autistic symptoms, the German translation [40] of the Autism-Spectrum-Quotient (AQ) [29] questionnaire was administered to each participant. The AQ comprises fifty statements, to which participants respond to with their degree of agreement (from “definitely disagree” to “definitely agree”). The total AQ score is composed of five subscales: social skills, attention switching, attention to detail, communication and imagination. These categories comprise five important domains which are commonly altered in autism spectrum disorders. Possible scores range from 0 to 50 points; the proposed cut-off lies at 32 points, although further diagnostics are necessary to warrant the diagnosis of ASD [29, 40].

### Stimulus material, task and procedure

The stimulus material comprised six sentences with high frequencies of use in everyday life. Two of them expressed a neutral („Ich bin ruhig”/ “I am calm”, „Ich bin etwas aufgeregt”/ “I am a bit excited”), two a positive („Ich fühle mich gut”/ “I feel good”, „Ich fühle mich großartig”/ “I feel great”) and two a negative („Ich fühle mich unwohl”/ “I feel uncomfortable”, „Ich fühle mich erbärmlich”/ “I feel awful”) emotional state. These six sentences were spoken by ten professional actors with a neutral, positive (happy) and negative (angry) prosody and facial expression and were recorded audiovisually. The nonverbal cues were tested for authenticity and included if authenticity reached an adequate score of at least 4 points on a 9-point-scale.

Previous studies have cautioned against including a large number of strongly incongruent stimuli, as this might bias participants' responding over the course of the testing procedure. Trimboli and Walker [41] reported that participants appeared to comprehend the study's intention when a large number of strongly incongruent stimuli were presented. Similarly, Olkoniemi and colleagues [42] found a shift in participant responding over the course of the experiment, in this case toward figurative rather than literal interpretations of sentences, which the authors attributed to the development of an expectation among participants that all stimuli contained figurative language. This expectation could be minimized by decreasing the percentage of strongly incongruent stimuli presented. Thus, in the current study, the final stimulus set comprised 40% congruent, 40% slightly incongruent and 20% strongly incongruent stimuli, for a total of 120 videos. Further information about creation and validation of this stimulus material can be found in Jacob et al., 2012 [43].

These 120 videos (mean duration = 1458 ms, *SD* = 316 ms) showed the faces of the 10 actors (5 women, 5 men; 12 videos each), speaking the short German sentences mentioned above. The combinations of different verbally and nonverbally expressed emotional states resulted in three groups of stimuli: Congruent (congruence between verbal and nonverbal message, e.g. positive_verbal_ + positive_nonverbal_; 48 out of 120 videos), slightly incongruent (positive/negative_verbal_ + neutral_nonverbal_ or positive/negative_nonverbal_ + neutral_verbal_; 48 out of 120 videos) and strongly incongruent (positive_verbal_ + negative_nonverbal_ or negative_verbal_ + positive_nonverbal_; 24 out of 120 videos) stimuli. Table 2 shows the composition of the stimulus material.

**Table 2:** Overview of the different combinations of stimuli (10).

Verbal valence	Nonverbal valence		
	Positive	Neutral	Negative
**Positive**	congruent (16)	slightly incongruent (12)	strongly incongruent (12)
**Neutral**	slightly incongruent (12)	congruent (16)	slightly incongruent (12)
**Negative**	strongly incongruent (12)	slightly incongruent (12)	congruent (16)

Note: Numbers in parentheses indicate the respective number of stimuli.

The experiment was performed on a computer with the program “Presentation” (Neurobehavioral Systems Inc, Albany, CA, USA). The volume was adjusted by every participant to an individually comfortable level. The sound was presented via headphones (Sennheiser HD 515, Sennheiser electronic GmbH & Co. KG, Wedemark, Wennebostel, Germany). The 120 videos were split into two blocks with 60 videos each, balanced for actors, sentences and congruence conditions, with stimulus order randomized within the blocks. The order of block presentation was reversed for half of the participants. The participants were asked to select the category they felt best matched their impression of the speaker’s expression. We used a forced-choice response format with the categories “happy”, “angry”, “ironic” and “ambivalent”. Each response category was defined and discussed in detail with participants to ensure similar interpretation of categories among study participants (see Table 3). As basic, relevant emotions with a high degree of arousal we offered “happy” and “angry” as positive and negative emotional categories [44–46]. The category “ambivalent” served as an alternative to avoid the participant choosing the “ironic” category only due to the absence of other response options when categorizing incongruent stimuli. Participants were instructed that the category “ambivalent” denominates an emotional state which genuinely expresses mixed feelings, e.g. being simultaneously happy and angry, while the term “ironic” marks an utterance with the additional intention to express something other, or the opposite of, what is said literally. Thus, “ironic” is the only category to contain information about the intention of the speaker in addition to the speaker’s emotional state [10].

**Table 3:** Categories and their definitions used in the experiment [10].

Category	Definition
**„ärgerlich“/“angry”**	„Der/die Sprecher/in drückt einen negativen Gefühlszustand aus. Er/sie ist schlecht gelaunt, ärgerlich.“
	“The speaker is expressing a negative emotional state. He/she is bad-tempered, angry.”
**„freudig“/“happy”**	„Der/die Sprecher/in drückt einen positiven Gefühlszustand aus. Er/sie ist gut gelaunt, fröhlich.“
	“The speaker is expressing a positive emotional state. He/she is good-tempered, happy.”
**„ironisch“/“ironic”**	„Der/die Sprecher/in verstellt sich, aber er/sie erwartet, dass die wahre Bedeutung seiner/ihrer Äußerung verstanden wird. Die Verstellung wird dabei eingesetzt, um eine besondere Wirkung zu erreichen.“
	“The speaker’s verbal description differs from his/her real emotional state, but he/she expects that the true meaning of his/her expression will be understood. This mode of expression is used to produce a particular effect.”
**„zwiespältig“/“ambivalent”**	„Der/die Sprecher/in drückt einen gemischten Gefühlszustand aus. Er/sie erlebt gleichzeitig widersprüchliche Gefühle, die beide vermittelt werden.“
	“The speaker is expressing a mixed emotional state. He/she is experiencing concurrent contradictory feelings that are both conveyed.”

To enter the respective classification, a Cedrus RB-730 Response Pad (Cedrus Corporation, San Pedro, CA, USA) was used. The order of the four response categories from left to right was varied among participants: Out of 24 possible arrangements, 20 were used in each group. No arrangement was used more than once in one group. Participants had five seconds to choose a category, beginning with the start of video presentation. Before the experiment started, a test run containing 10 videos which were not used in the main experiment was conducted to accustom the participants to the Response Pad and the task [10].

### Data analysis

The data were analyzed with the software IBM SPSS Statistics Version 23 (IBM Corporation, Armonk, NY, USA). The categorical ratings were transformed to choice frequencies. Choice frequency of each category (“angry”, “happy”, “ironic” and “ambivalent”) and reaction times were calculated separately for each congruence condition.

To evaluate our hypotheses, we conducted a two factorial ANOVA with group as a between subject factor (ASD and TD group) and congruence condition as a within subject factor (congruent and incongruent). Significant effects (considering a statistical threshold of p < 0.05) were further evaluated using post hoc t-tests. Moreover, the impact of the degree of incongruency (congruent, slightly incongruent, strongly incongruent) on the choice frequency for the category "ironic" was assessed in more detail in a further explorative analysis using t-tests, and Cohen’s *d* for a calculation of effect size.

In an additional explorative analysis, another two factorial ANOVA was conducted to evaluate the effects of group and congruence condition on reaction times. Lastly, the impact of symptom severity (as measured by AQ scores) on the choice frequency for the “ironic” category and the respective reaction times was calculated by a correlational analysis using Spearman’s Rho, due to non-normally distributed data in the AQ subscores as indicated by the Kolmogorov-Smirnov-test.

## Results

### Choice frequency of the category “ironic”

We found a significant main effect for “congruence condition” on the choice frequency for the “ironic” category (ANOVA: F(1, 38) = 127.3, *p* < 0.001, *η*^*2*^ = 0.77). A post-hoc t-test revealed that incongruent stimuli were categorized as "ironic" significantly more often than congruent stimuli (incongruent: *M* = 25.8%, *SD* = 9.0%; congruent: *M* = 8.0%, *SD* = 6.8%; *T* = -10.8, *p*< 0.001) across all participants.

**Fig 1:**
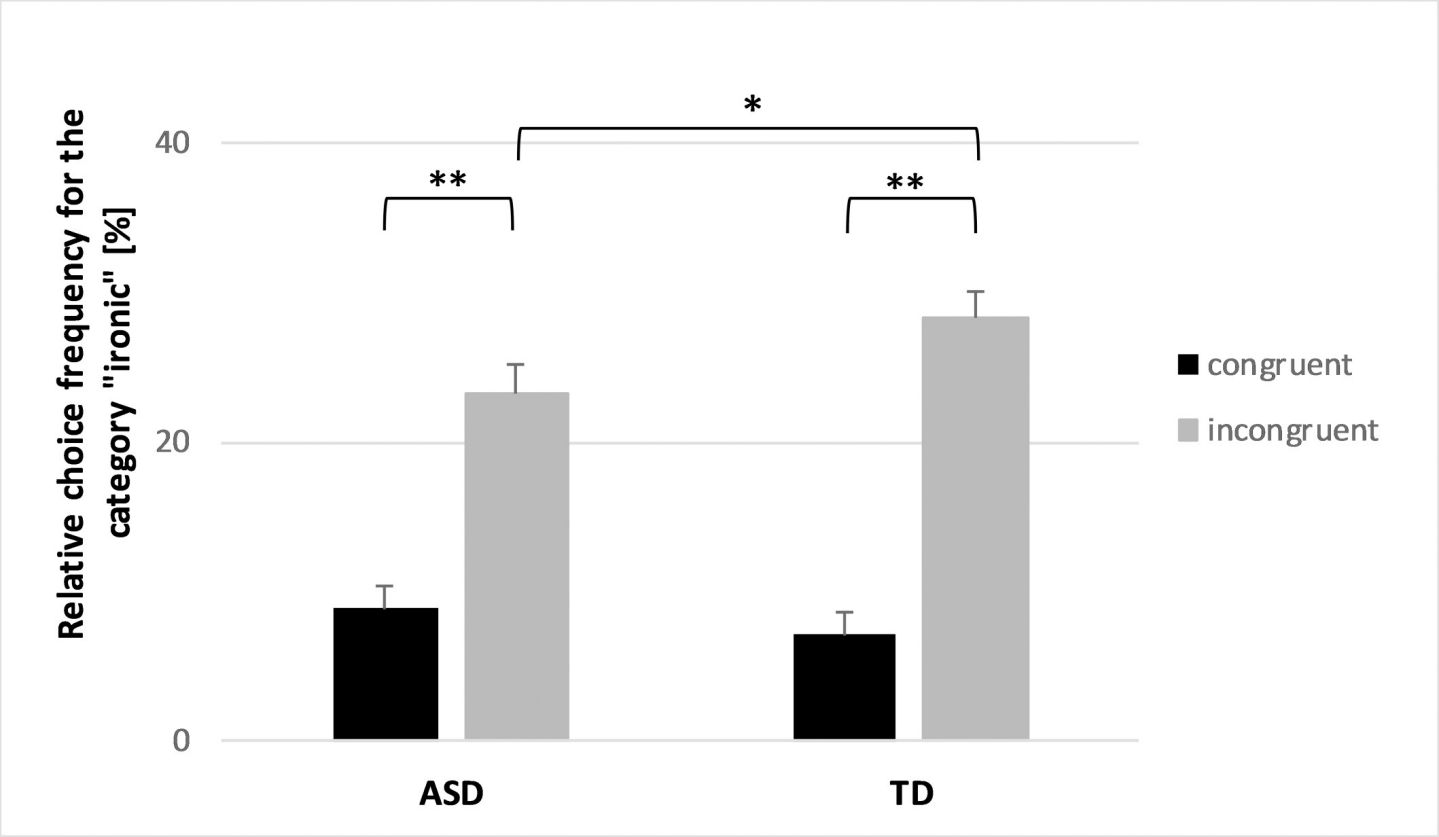
Relative choice frequencies for the category “ironic” for both groups under the different congruence conditions. Within each group, incongruent stimuli were categorized significantly more frequently as "ironic" than were congruent stimuli (** *p* < 0.01). For incongruent stimuli, we found a significantly lower choice frequency in the ASD group compared to the TD group (* *p* = 0.038). The bars indicate the means with standard errors.

The ANOVA did not reveal a significant difference between the ASD group and the TD group across all stimuli (F(1, 38) = 0.8, *p* = 0.39, *η*^*2*^ = 0.02). However, a significant interaction for “congruence condition” and “group” was observed (F(1, 38) = 4.6, *p* = 0.038, *η*^*2*^ = 0.11). Post-hoc tests revealed a significant lower choice frequency for the category “ironic” for incongruent stimuli in ASD subjects as compared to the TD group (ASD: *M* = 23.2%, *SD* = 8.8%; TD: *M* = 28.3%, *SD* = 8.2%; *p* = 0.04, *d* = 0.58), but no significant differences for congruent stimuli (ASD: *M* = 8.8%, *SD* = 6.6%; TD: *M* = 7.1%, *SD* = 6.7%; *p* = 0.21, *d* = -0.25). In Fig 1, the relative choice frequency of the category “ironic” is shown for both groups and congruence conditions.

The choice frequencies for each of the four different categories (“angry”, “happy”, “ironic” and “ambivalent”) for the ASD group and the TD group are presented as supplementary data (see S1 Table).

Regarding the more detailed evaluation of group differences in irony attribution for the three different congruency conditions (congruent, slightly incongruent and strongly incongruent) the explorative analysis revealed that subjects with ASD classified strongly incongruent stimuli less frequently as "ironic" than TD controls (*p* = 0.02, *d* = 0.66). For congruent (*p* = 0.21, *d* = -0.25) and slightly incongruent stimuli (*p* = 0.28, *d* = 0.19)–in contrast–no group differences in irony attribution were found (see S1 Table).

### Reaction times

The categorization of incongruent stimuli as expressing irony was associated with shorter reaction times as compared to the categorization of congruent stimuli as expressing irony (F(1, 33) = 5.8, *p* = 0.02, *η^2^* = 0.15). However, the ANOVA did neither reveal a main effect of group (F(1,33) = 0.2, *p* = 0.64, *η^2^* = 0.01), nor a significant interaction between group and congruence condition on reaction times (F(1, 33) = 0.3, *p* = 0.60, *η^2^* = 0.01). Reaction times for the ASD group and the TD group are presented for each of the four categories ("angry", "happy", "ironic" and "ambivalent") as supplementary data (see S1 Text).

### AQ scores

In the TD group, the total AQ score ranged from 5 to 20 points (mean score = 11.5, *SD* = 4.1), while, in the ASD group, the range was 20 to 48 points (mean score = 38.3, *SD* = 7.2).

### Correlation of AQ score and “ironic” choice frequency

We did not find any correlation between AQ scores or its subscales and choice frequency for the category “ironic” in any congruence condition (all abs(*r*) < 0.26; all *p* > 0.05; two-tailed).

## Discussion

The aim of the study was to evaluate differences in the impression of irony created by the mismatch of verbal and nonverbal cues in patients with ASD as compared to typically developed subjects. We hypothesized that the incongruence between verbal and nonverbal emotional cues is perceived as expressing irony across all participants. This hypothesis was confirmed, in line with Jacob and colleagues’ previous findings in typically developed subjects [10].

Regarding group differences, subjects with ASD classified incongruent stimuli significantly less frequently as expressing irony than TD controls. This finding is in accordance with our second hypothesis and with several other studies reporting an impaired comprehension of irony in adults with ASD [16–24]. Regarding the former studies’ tasks, it is apparent that mentalizing processes of different degrees, or orders, are necessary for their completion [21]. In this context, mentalizing processes refer to inferences about a person’s state of mind. The importance of these processes for pragmatic language understanding has already been shown for both ASD [47, 48] and TD [49] individuals. Mentalizing processes can be considered in multiple orders: First-order mentalizing processes answer the question “What does person A think, believe or feel?” Using first-order mentalizing, a concept of the speaker’s mental state is formed, which might lead to the conclusion that the verbally and nonverbally communicated mental states do not match. In case of a mismatch first-order mentalizing might lead to the conclusion that either the verbal or the nonverbal component is a more trustworthy indicator of the true emotional state, or that both components are valid and the speaker is experiencing mixed feelings. Thus, in our model, first-order mentalizing processes might have led to an impression of anger, happiness or ambivalence for mismatched (incongruent) stimuli. While previous studies for which first-order mentalization was sufficient for completing the task partly showed differences between TD and ASD individuals in irony perception [17, 19, 21, 24], others reported no differences [31–33]. This heterogeneity might be explained by evidence that in ASD subjects, the tendency to use mentalizing in general might be diminished, but the tendency to use first-order mentalizing is relatively unimpaired compared with TD subjects [48, 50–54]. In accordance with this consideration, in our study the ASD group showed no differences to TD subjects in the classification of incongruent stimuli as belonging to specific emotional categories (“happy” or “angry”) or belonging to the “ambivalent” category.

Second-order mentalizing processes add another level (“What does person B think that person A thinks, believes or feels?). In our study, second-order mentalizing might lead to the conclusion that the speaker is explicitly implementing a verbal-nonverbal mismatch in the expectation that the addressee will understand the mismatch is intended to express a particular type of message, namely, an ironic one. Therefore, second-order mentalizing processes might have led to an impression of irony in our task. A tendency to implement higher order mentalizing processes may thus lead to the perception of irony in incongruent stimuli, while the implementation of only first-order mentalizing might lead to an impression of ambivalence, happiness or anger. Thus, in our study, the reduced impression of irony in ASD might be due to quantitative or qualitative differences in the tendency to implement higher or lower order mentalizing processes between TD and ASD [21]. Presumably TD subjects relied more on second-order mentalizing, therefore they perceived an impression of irony more often than ASD subjects, who have been shown to have a diminished tendency to use higher order mentalizing, but a relatively preserved tendency to rely on first-order mentalizing [53, 54]. This consideration is in line with the results of previous studies on irony perception: all studies requiring second-order mentalizing processes [16, 18, 22, 23] reported differences in irony perception between ASD and TD, while every study that did not report group differences [31–33] used tasks which could be completed using first-order mentalizing.

Differences between ASD and TD in irony attribution only occurred in strongly incongruent, but not in slightly incongruent or congruent stimuli. Considered differently, the influence of increasing mismatch on the impression of irony in the TD group was much larger than the corresponding influence on impression of irony in the ASD group. In the latter, the impression of irony also increased with increasing incongruency, but not as strongly. This might be because in stimuli with a greater mismatch, the influence of mentalizing processes on the impression of the speaker’s expression might be higher.

It is important to note that the actors were not asked to create an expression of irony but an authentic expression of an angry, happy or neutral emotional state at the nonverbal level combined with a sentence with a neutral, positive or negative emotional valence at the verbal level. There has not been a right or wrong way to classify the mismatch of verbal and nonverbal cues. Instead, we measured the tendency how far this mismatch is interpreted as expressing irony. This tendency was more pronounced in the TD group and in stimuli with a stronger incongruency.

The present findings should be interpreted cautiously due to the relatively small sample size. In addition, during the experiment, participants had to choose among four predefined categories under forced choice conditions. However, some participants reported difficulties in choosing a suitable category, especially in stimuli with neutral nonverbal expressions. Further investigations using additional response categories such as “neutral” or “dishonest” could therefore be useful to increase sensitivity between ironic intent attribution and attribution of other emotional states, attitudes, intentions, or other personal attributes of the speaker (see also Jacob and colleagues [10]).

## Conclusions

Our results indicate that incongruent verbal and nonverbal signals create an impression of irony significantly less often in participants with high-functioning autism than in typically developed control subjects. Since the extent of overall autistic symptoms did not correlate with the reduced tendency to attribute incongruent stimuli as expressing irony, the attenuation in irony attribution might rather be related to specific subdomains of autistic traits, such as a reduced tendency to interpret communicative signals in terms of complex intentional mental states. The observed differences in irony attribution support the assumption that a less pronounced tendency to engage in higher order mentalization processes might underlie the impairment of pragmatic language understanding in high functioning autism. Further research is necessary to evaluate the effect of mentalizing abilities on intent attribution and pragmatic language understanding in autism spectrum disorder.

## Supporting information

S1 File Overview of choice frequencies and reaction times for each participant. (CSV)

S1 Table Summary of choice frequencies. Choice frequencies are given in percent.
(PDF)

S1 Text Explorative analysis of reaction times. (PDF)

## References

[1] Gluksberg S. Understanding Figurative Language: From Metaphor to Idioms. New York: Oxford University Press; 2001.

[2] Gibbs RW Jr, Colston HL. Chapter 21—Figurative Language A2—Traxler, Matthew J In: Gernsbacher MA, editor. Handbook of Psycholinguistics (Second Edition). London: Academic Press; 2006 p. 835–61.

[3] Irony (n.d.) In Merriam-Webster.com. Retrieved March 15, 2017, from http://www.merriam-webster.com/dictionary/irony.

[4] Dews S, Kaplan J, Winner E. Why not say it directly? The social functions of irony. Discourse Processes. 1995;19(3):347–67. 10.1080/01638539509544922

[5] Dews S, Winner E. Muting the Meaning A Social Function of Irony. Metaphor and Symbolic Activity. 1995;10(1):3–19. 10.1207/s15327868ms1001_2

[6] Filik R, Brightman E, Gathercole C, Leuthold H. The emotional impact of verbal irony: Eye-tracking evidence for a two-stage process. Journal of Memory and Language. 2017;93:193–202. 10.1016/j.jml.2016.09.006.

[7] Levinson SC. Pragmatics. [Nachdr.] ed. Cambridge [u.a.]: Cambridge Univ. Press; 2011 XVI, 420 S. p.

[8] Jorgensen J. The functions of sarcastic irony in speech. Journal of Pragmatics. 1996;26(5):613–34. 10.1016/0378-2166(95)00067-4.

[9] Rockwell P. Lower, Slower, Louder: Vocal Cues of Sarcasm. Journal of Psycholinguistic Research. 2000;29(5):483–95. 10.1023/a:1005120109296

[10] Jacob H, Kreifelts B, Nizielski S, Schütz A, Wildgruber D. Effects of Emotional Intelligence on the Impression of Irony Created by the Mismatch between Verbal and Nonverbal Cues. PloS one. 2016;11(10):e0163211 Epub 2016/10/08. 10.1371/journal.pone.0163211 ; PubMed Central PMCID: PMCPMC5055337. 27716831 PMC5055337

[11] Kreuz RJ, Roberts RM, Johnson BK, Bertus EL. Figurative language occurrence and co-occurrence in contemporary literature In: Kreuz RJ, MacNealy MS, Kreuz RJ, MacNealy MS, editors. Empirical approaches to literature and aesthetics. Advances in discourse processes, Vol. 52 Westport, CT, US: Ablex Publishing; 1996 p. 83–97.

[12] Schwoebel J, Dews S, Winner E, Srinivas K. Obligatory Processing of the Literal Meaning of Ironic Utterances: Further Evidence. Metaphor & Symbol. 2000;15(1/2):47–61. PubMed PMID: 3331787.

[13] Gibbs RW. Irony in Talk Among Friends. Metaphor and Symbol. 2000;15(1–2):5–27. 10.1080/10926488.2000.9678862

[14] Varga E, Simon M, Tenyi T, Schnell Z, Hajnal A, Orsi G, et al Irony comprehension and context processing in schizophrenia during remission—a functional MRI study. Brain Lang. 2013;126(3):231–42. Epub 2013/07/23. 10.1016/j.bandl.2013.05.017 . 23867921

[15] Amenta S, Noel X, Verbanck P, Campanella S. Decoding of emotional components in complex communicative situations (irony) and its relation to empathic abilities in male chronic alcoholics: an issue for treatment. Alcoholism, clinical and experimental research. 2013;37(2):339–47. Epub 2012/11/10. 10.1111/j.1530-0277.2012.01909.x . 23136931

[16] Channon S, Crawford S, Orlowska D, Parikh N, Thoma P. Mentalising and social problem solving in adults with Asperger's syndrome. Cognitive neuropsychiatry. 2014;19(2):149–63. Epub 2013/07/24. 10.1080/13546805.2013.809659 ; PubMed Central PMCID: PMCPMC4095949. 23875885 PMC4095949

[17] Huang SF, Oi M, Taguchi A. Comprehension of figurative language in Taiwanese children with autism: The role of theory of mind and receptive vocabulary. Clinical linguistics & phonetics. 2015;29(8–10):764–75. Epub 2015/04/25. 10.3109/02699206.2015.1027833 . 25909823

[18] Li JP, Law T, Lam GY, To CK. Role of sentence-final particles and prosody in irony comprehension in Cantonese-speaking children with and without Autism Spectrum Disorders. Clinical linguistics & phonetics. 2013;27(1):18–32. Epub 2012/12/15. 10.3109/02699206.2012.734893 . 23237415

[19] Adachi T, Koeda T, Hirabayashi S, Maeoka Y, Shiota M, Wright EC, et al The metaphor and sarcasm scenario test: a new instrument to help differentiate high functioning pervasive developmental disorder from attention deficit/hyperactivity disorder. Brain & development. 2004;26(5):301–6. Epub 2004/05/29. 10.1016/s0387-7604(03)00170-0 . 15165670

[20] Saban-Bezalel R, Mashal N. Hemispheric Processing of Idioms and Irony in Adults With and Without Pervasive Developmental Disorder. Journal of autism and developmental disorders. 2015;45(11):3496–508. Epub 2015/06/14. 10.1007/s10803-015-2496-4 . 26070277

[21] Deliens G, Papastamou F, Ruytenbeek N, Geelhand P, Kissine M. Selective Pragmatic Impairment in Autism Spectrum Disorder: Indirect Requests Versus Irony. Journal of autism and developmental disorders. 2018 Epub 2018/04/11. 10.1007/s10803-018-3561-6 . 29633109

[22] Mathersul D, McDonald S, Rushby JA. Understanding advanced theory of mind and empathy in high-functioning adults with autism spectrum disorder. Journal of clinical and experimental neuropsychology. 2013;35(6):655–68. Epub 2013/06/27. 10.1080/13803395.2013.809700 . 23799244

[23] Scholten I, Engelen E, Hendriks P. Understanding Irony in Autism: The Role of Context and Prosody In: Ghosh S, Szymanik J, editors. The Facts Matter Essays on Logic and Cognition in Honour of Rineke Verbrugge. London: College Publications; 2015 p. 121–32.

[24] Williams DL, Cherkassky VL, Mason RA, Keller TA, Minshew NJ, Just MA. Brain function differences in language processing in children and adults with autism. Autism Res. 2013;6(4):288–302. Epub 2013/03/16. 10.1002/aur.1291 ; PubMed Central PMCID: PMCPMC4492467. 23495230 PMC4492467

[25] American Psychiatric Association. Diagnostic and Statistical Manual of Mental Disorders. Washington, DC2013.

[26] Rutherford MD, Baron-Cohen S, Wheelwright S. Reading the mind in the voice: a study with normal adults and adults with Asperger syndrome and high functioning autism. Journal of Autism & Developmental Disorders. 2002;32(3):189–94. . 12108620 10.1023/a:1015497629971

[27] Stewart ME, McAdam C, Ota M, Peppe S, Cleland J. Emotional recognition in autism spectrum conditions from voices and faces. Autism. 2013;17(1):6–14. Epub 2012/10/10. 10.1177/1362361311424572 . 23045218

[28] Wing L. The autistic continuum In: Wing L, editor. Aspects of autism: Biological research. London: Gaskell/Royal College of Psychiatrists; 1988.

[29] Baron-Cohen S, Wheelwright S, Skinner R. The Autism-spectrum quotient (AQ): evidence from Asperger syndrome/high-functioning autism, males and females, scientists and mathematicians. Journal of Autism & Developmental Disorders. 2001;31(1):5–17. . 11439754 10.1023/a:1005653411471

[30] Lindell AK, Notice K, Withers K. Reduced language processing asymmetry in non- autistic individuals with high levels of autism traits. Laterality: Asymmetries of Body, Brain and Cognition. 2009;14(5):457–72. 10.1080/13576500802507752 19051130

[31] Au-Yeung SK, Kaakinen JK, Liversedge SP, Benson V. Processing of Written Irony in Autism Spectrum Disorder: An Eye-Movement Study. Autism Res. 2015;8(6):749–60. 10.1002/aur.1490 . 25962666

[32] Glenwright M, Agbayewa AS. Older children and adolescents with high-functioning autism spectrum disorders can comprehend verbal irony in computer-mediated communication. Research in Autism Spectrum Disorders. 2012;6(2):628–38. 10.1016/j.rasd.2011.09.013.

[33] Colich NL, Wang AT, Rudie JD, Hernandez LM, Bookheimer SY, Dapretto M. Atypical Neural Processing of Ironic and Sincere Remarks in Children and Adolescents with Autism Spectrum Disorders. Metaphor Symb. 2012;27(1):70–92. Epub 2012/01/01. 10.1080/10926488.2012.638856 ; PubMed Central PMCID: PMCPMC3909704. 24497750 PMC3909704

[34] Baron-Cohen S, Wheelwright S. The Empathy Quotient: An Investigation of Adults with Asperger Syndrome or High Functioning Autism, and Normal Sex Differences. Journal of autism and developmental disorders. 2004;34(2):163–75. 10.1023/b:jadd.0000022607.19833.00 15162935

[35] Lehrl S. Mehrfachwahl-Wortschatz-Intelligenztest MWT-B. Balingen: Spitta Verlag; 2005

[36] Beck AT, Ward CH, Mendelson M, Mock J, Erbaugh J. An inventory for measuring depression. Archives of general psychiatry. 1961;4:561–71. Epub 1961/06/01. . 13688369 10.1001/archpsyc.1961.01710120031004

[37] Constantino J, Gruber C. Social Responsiveness Scale (SRS). Los Angeles: Western Psychological Services; 2005.

[38] Rutter M, Bailey A, Lord C. The Social Communication Questionnaire. Los Angeles: Western Psychological Services; 2003.

[39] Kamp-Becker I, Mattejat F, Wolf-Ostermann K, Remschmidt H. Die Marburger Beurteilungsskala zum Asperger-Syndrom (MBAS)—ein Screening-Verfahren für autistische Störungen auf hohem Funktionsniveau. Zeitschrift für Kinder- und Jugendpsychiatrie und Psychotherapie. 2005;33(1):15–26. 10.1024/1422-4917.33.1.15 . 15714837

[40] Freitag CM, Retz-Junginger P, Retz W, Seitz C, Palmason H, Meyer J, et al Evaluation der deutschen Version des Autismus-Spektrum-Quotienten (AQ)—die Kurzversion AQ-k. Zeitschrift für Klinische Psychologie und Psychotherapie. 2007;36(4):280–9. 10.1026/1616-3443.36.4.280

[41] Trimboli A, Walker MB. Nonverbal dominance in the communication of affect: A myth? Journal of Nonverbal Behavior. 1987;11(3):180–90. 10.1007/bf00990236

[42] Olkoniemi H, Ranta H, Kaakinen JK. Individual differences in the processing of written sarcasm and metaphor: Evidence from eye movements. Journal of experimental psychology Learning, memory, and cognition. 2016;42(3):433–50. Epub 2015/09/16. 10.1037/xlm0000176 . 26371496

[43] Jacob H, Kreifelts B, Brück C, Erb M, Hösl F, Wildgruber D. Cerebral integration of verbal and nonverbal emotional cues: Impact of individual nonverbal dominance. NeuroImage. 2012;61(3):738–47. 10.1016/j.neuroimage.2012.03.085. 22516367

[44] Ekman P. What Scientists Who Study Emotion Agree About. Perspectives on Psychological Science. 2016;11(1):31–4. 10.1177/1745691615596992 . 26817724

[45] Shaver P, Schwartz J, Kirson D, O'Connor C. Emotion knowledge: Further exploration of a prototype approach. Journal of Personality and Social Psychology. 1987;52(6):1061–86. 10.1037/0022-3514.52.6.1061 3598857

[46] Russell JA. A circumplex model of affect. Journal of Personality and Social Psychology. 1980;39(6):1161–78. 10.1037/h0077714

[47] Happe FG. Communicative competence and theory of mind in autism: a test of relevance theory. Cognition. 1993;48(2):101–19. Epub 1993/08/01. . 8243028 10.1016/0010-0277(93)90026-r

[48] Happé FGE. Understanding Minds and Metaphors: Insights from the Study of Figurative Language in Autism. Metaphor & Symbolic Activity. 1995;10(4):275. PubMed PMID: 7315389.

[49] Sullivan K WE, Hopfield N. How children tell a lie from a joke: The role of second- order mental state attributions. British Journal of Developmental Psychology. 1995;13(2):191–204. 10.1016/j.conb.2010.08.014 PubMed PMID: 20832291.

[50] Baron-Cohen S. Theory of mind in normal development and autism. Prisme. 2001;34:174–83.

[51] Leekam SR, Perner J. Does the autistic child have a metarepresentational deficit? Cognition. 1991;40(3):203–18. Epub 1991/09/01. . 1786675 10.1016/0010-0277(91)90025-y

[52] Yirmiya N, Solomonica-Levi D, Shulman C, Pilowsky T. Theory of mind abilities in individuals with autism, Down syndrome, and mental retardation of unknown etiology: the role of age and intelligence. Journal of child psychology and psychiatry, and allied disciplines. 1996;37(8):1003–14. Epub 1996/11/01. . 9119934 10.1111/j.1469-7610.1996.tb01497.x

[53] Martin I, McDonald S. An exploration of causes of non-literal language problems in individuals with Asperger Syndrome. Journal of autism and developmental disorders. 2004;34(3):311–28. Epub 2004/07/22. . 15264499 10.1023/b:jadd.0000029553.52889.15

[54] Happe FG. An advanced test of theory of mind: understanding of story characters' thoughts and feelings by able autistic, mentally handicapped, and normal children and adults. Journal of autism and developmental disorders. 1994;24(2):129–54. Epub 1994/04/01. . 8040158 10.1007/BF02172093

