# Supplementary material for: Attenuated impression of irony created by the mismatch of verbal and nonverbal cues in patients with autism spectrum disorder
Source: PLoS One. 2018 Oct 15;13(10):e0205750. doi: 10.1371/journal.pone.0205750 (PMC6188779; doi:10.1371/journal.pone.0205750)
Supplement: S1 Table — Choice frequencies are given in percent. (PDF) [file pone.0205750.s002.pdf]

S1 Table. Summary of choice frequencies.

Choice frequency	TD		ASD		*t*-value	*p*-value (one-tailed)	Cohen's *d*
	M	***SD***	***M***	***SD***			
Total							
Angry	29.9	8.7	33.8	8.6			
Happy	22.9	6.0	21.9	4.2			
Ironic	19.8	6.0	17.5	6.4	1.17	0.13	0.37
Ambivalent	27.4	8.8	26.8	8.0			
Congruent							
Angry	35.4	7.6	40.0	9.9			
Happy	31.1	6.6	29.8	5.7			
Ironic	7.1	6.7	8.8	6.6	-0.80	0.21	-0.25
Ambivalent	26.5	7.7	21.4	8.6			
Incongruent (Total)							
Angry	26.3	10.7	29.7	8.5			

Happy	17.5	7.0	16.6	5.8			
Ironic	28.3	8.2	23.2	8.8	1.84	*0.04**	0.58
Ambivalent	28.0	10.7	30.5	10.6			
Slightly incongruent							
Angry	31.0	12.1	34.1	11.4			
Happy	20.7	7.5	19.1	4.8			
Ironic	17.2	8.1	15.6	8.9	0.59	0.28	0.19
Ambivalent	31.1	12.3	31.2	10.0			
Strongly incongruent							
Angry	17.0	13.0	21.1	11.4			
Happy	10.9	14.4	11.5	12.3			
Ironic	50.5	18.4	38.5	17.3	2.07	*0.02**	0.66
Ambivalent	21.6	12.9	28.9	19.6			

Choice frequencies are given in percent.

* The asterisk marks a significant difference.
