# Supplementary material for: Attenuated impression of irony created by the mismatch of verbal and nonverbal cues in patients with autism spectrum disorder
Source: PLoS One. 2018 Oct 15;13(10):e0205750. doi: 10.1371/journal.pone.0205750 (PMC6188779; doi:10.1371/journal.pone.0205750)
Supplement: S1 Text — (PDF) [file pone.0205750.s003.pdf]

Explorative Analysis: reaction times

An explorative analysis was performed to compare reaction times of stimuli which were categorized as "ironic". Since not every participant categorized at least one stimulus as "ironic" in every congruence condition, a total of 16 TD participants and 19 ASD participants were included in this analysis.

We conducted a 2 x 2 ANOVA with group as a between subject factor (ASD and TD group) and congruence condition as a within subject factor (congruent and incongruent) to evaluate reaction times for "ironic" responses.

We found a significant main effect for congruence condition (F(1, 33) = 5.8, p = 0.02, η^2 = 0.15), with faster reaction times for incongruent than congruent stimuli that were categorized as “ironic”. We did not find a significant main effect for group (F(1,33) = 0.2, p = 0.64, η^2 = 0.01) nor a significant interaction (F(1, 33) = 0.3, p = 0.60, η^2 = 0.01).

For incongruent stimuli, the mean reaction time for “ironic” was 3,144 ms (SD = 354 ms) in the ASD group and 2,988 ms (SD = 521 ms) in the TD group. Taking the degree of incongruence into account, the shortest reaction times for the “ironic” category were measured for the strongly incongruent stimuli (ASD: *M* = 3,083 ms, *SD* = 79 ms; TD: *M* = 2,960 ms, *SD* = 119 ms), followed by the slightly incongruent stimuli (ASD: *M* = 3,191 ms, *SD* = 97 ms; TD: *M* = 3,061 ms, *SD* = 132 ms). The longest reaction times were found for the congruent stimuli (ASD: *M* = 3,322 ms, *SD* = 156 ms; TD: *M* = 3,296 ms, *SD* = 175 ms).

Table A. Summary of reaction times.

Reaction time	TD		ASD	
	M	***SD***	***M***	***SD***
Total				
Angry	2,666	480	2,664	310
Happy	2,346	431	2,332	321
Ironic	3,002	508	3,160	367
Ambivalent	3,168	539	3,169	331
Congruent				
Angry	2,570	423	2,592	265
Happy	2,195	451	2,222	331
Ironic	3,296	702	3,322	680
Ambivalent	3,215	595	3,301	383
Incongruent (Total)				
Angry	2,765	546	2,721	372
Happy	2,560	503	2,461	345
Ironic	2,988	521	3,144	354
Ambivalent	3,125	504	3,123	340
Slightly incongruent				
Angry	2,816	548	2,733	365
Happy	2,496	504	2,398	300
Ironic	3,061	588	3,191	433
Ambivalent	3,148	530	3,161	363
Strongly incongruent				

Angry	2,676	721	2,700	441
Happy	2,852	892	2,622	586
Ironic	2,960	532	3,083	355
Ambivalent	3,068	480	3,067	330

Times are given in milliseconds.
